# Size dependent antipredator responses in a fish–shrimp mutualism

**DOI:** 10.1098/rsbl.2023.0285

**Published:** 2024-03-13

**Authors:** Giovanni Polverino, Topi K. Lehtonen, Andrew Geschke, Tara Callahan, Jessica Urbancic, Bob B. M. Wong

**Affiliations:** ^1^ School of Biological Sciences, Monash University, Melbourne, Australia; ^2^ Department of Ecological and Biological Sciences, University of Tuscia, Viterbo, Italy; ^3^ Centre for Evolutionary Biology, School of Biological Sciences, University of Western Australia, Perth, Western Australia, Australia; ^4^ Department of Biology, University of Turku, Turku, Varsinais-Suomi, Finland

**Keywords:** antipredator behaviour, body size, predation risk, refuge use, risk perception, optimal strategy

## Abstract

For prey, taking refuge from predators has obvious fitness benefits but may also be costly by impinging on time and effort available for feeding or attracting mates. The antipredator responses of refuge-seeking animals are therefore predicted to vary strategically depending on how threatening they perceive the risk. To test this, we studied the impacts of a simulated predatory threat on the antipredator responses of wild sandy prawn-gobies (*Ctenogobiops feroculus*) that co-inhabit burrows with *Alpheus* shrimp (family Alpheidae) in a mutualistic relationship. We exposed goby–shrimp pairs, repeatedly on three separate occasions, to an approaching threat and measured the antipredator behaviours of both partners. We found that re-emerging from the burrow took longer in large compared to small fish. Moreover, quicker re-emergence by small—but not medium or large-sized gobies—was associated with an earlier flight from the approaching threat (i.e. when the threat was still further away). Finally, the goby and shrimp sharing a burrow were matched in body size and their risk-taking behaviour was highly dependent on one another. The findings contribute to our understanding of how an individual's phenotype and perception of danger relates to its risk-taking strategy, and how mutualistic partners can have similar risk sensitivities.

## 1. Introduction

Time and effort spent on predator detection and avoidance can be critical to survival. However, such activities can also conflict with other important behaviours, such as foraging and mate attraction [1–3]. Indeed, the energetic demands of antipredator behaviours can be substantial enough to have negative population and ecosystem-level consequences [4–7]. Theory predicts that individuals should be able to behave in a risk-sensitive manner and adjust their antipredator responses to the actual level of a threat, while minimizing the associated costs [8–11]. This is especially important for refuge-seeking species, for which prolonged time spent hiding may not only impinge on behaviours that are critical to fitness, but also reduce their ability to acquire further information about the threat [12,13].

Within species, risk perception is expected to vary among individuals. For instance, large and small individuals may differ in their vulnerability to predators, which, in turn, can affect their risk perception and, thus, their optimal strategies and behavioural responses [14,15]. For instance, an individual's value as prey to would-be predators may depend on its size; larger individuals can be more susceptible when they provide greater energetic returns [16,17], while they tend to be less vulnerable to gape-size limited predators [18]. Size-dependent metabolic demands may also influence antipredator behaviours, such as refuge use, when the costs of hiding—such as lost feeding opportunities—are better sustained by large than small individuals, as has been shown in three-spined sticklebacks, *Gasterosteus aculeatus* [14].

Between species, individuals often respond to the antipredator behaviours of heterospecifics. Many animals, for example, eavesdrop on the antipredator alarm calls of other species to alert them of danger (reviewed in [19]). Particularly sophisticated antipredator behaviours between species can even promote more permanent mutualisms. Here, the partners in the relationship can potentially differ in their vulnerabilities, and hence, their risk perception, to predatory threats. Such vulnerability differences between species are likely to influence their behavioural dynamics.

Certain burrow-dwelling marine fish live in an intriguing mutualism with shrimp. For example, in coral reefs around the world, over 120 different species of gobies and 20 alpheid shrimp are known to engage in mutualistic interactions [20]. The shrimp constructs a burrow in the sandy substrate on the seabed, which acts as a refuge against would-be predators and thus benefits the goby, while the visual acuity and vigilance of the goby benefit the shrimp by warning it of impending threats [21–23] (see also electronic supplementary material, Appendix 1).

We set out to investigate risk-perception and antipredator behaviour in burrow-dwelling sandy prawn-gobies (*Ctenogobiops feroculus*) and shrimp (family Alpheidae). To do so, we repeatedly exposed gobies and shrimp to an approaching threat. We focused on ecologically-relevant behaviours typically associated with risk-sensitivity in refuge-seeking species, specifically the distance at which the focal animals fled when approached by the threat (i.e. the flight-initiation distance; FID) and the latency to re-emerge from their burrow after the threat (RET) [14,24]. We also included body size and environmental data (e.g. time of the day) in the analysis, given the importance of body size and ecological conditions in influencing risk-taking behaviours [14,15,25,26]. Here, we were especially interested in testing the hypothesis that body size could potentially mediate the relationship between FID and RET.

## 2. Material and methods

### (a) Study location

We conducted the study at Heron Island (23°27′ S, 151°55′ E) on the Great Barrier Reef, Australia. Experiments were carried out between approximately 06.00–18.00 in shallow waters 20–70 m from the shoreline at water depths of 0.3–1.2 m. Trials were carried out on single goby–shrimp pairs sharing the same burrow. While multiple gobies or shrimp can sometimes share the same burrow, such partnerships were infrequent at the time of our study. As a result, and because we wanted to control for any potential density effects that might arise from differences in the number and composition of the burrows' occupants, we excluded burrows that housed multiple gobies or shrimp.

### (b) Experimental procedure

**Figure 1.**
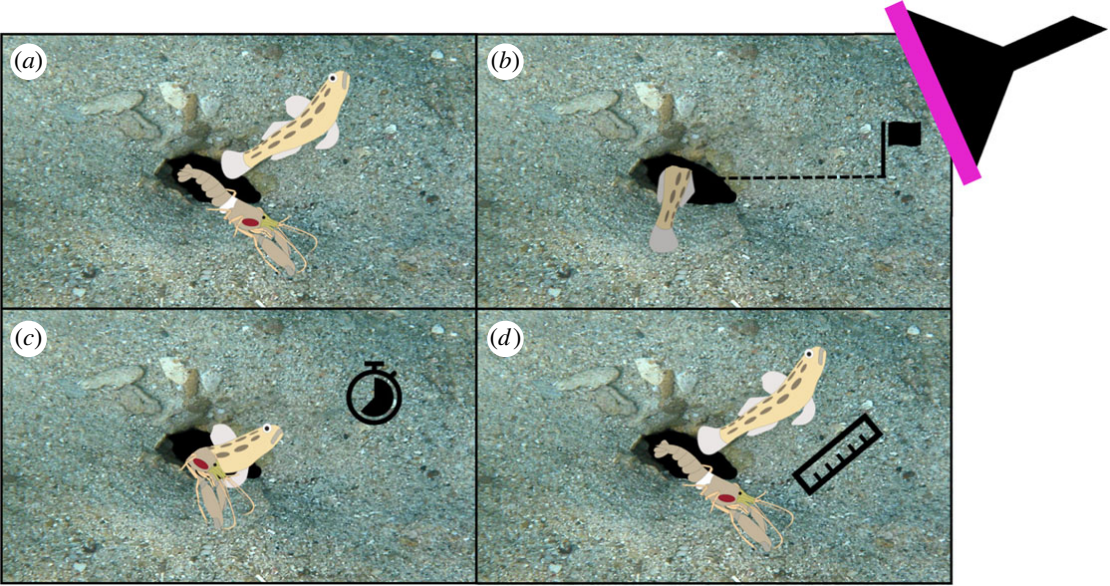
Graphics of the antipredator responses assayed: (*a*) goby–shrimp pair; (*b*) flight-initiation distance (FID) measured when the pair retreated in response to an approaching novel threat (i.e. probe); (*c*) re-emergence time (RET) up to 20 min; and (*d*) body size of the goby and shrimp measured after the assay.

To assess the risk-sensitivity of the focal animals, we exposed each focal goby–shrimp pair to a simulated threat on three separate occasions. To do so, we first located a goby–shrimp pair. Observers (i.e. snorkellers) then positioned themselves 2 m from the burrow to reduce disturbance and waited for the focal goby and shrimp to re-emerge from their burrow and commence routine behaviours (i.e. feeding, sand shifting, burrow maintenance; figure 1*a* and electronic supplementary material, Appendix 1). After a further 2-min period, an observer moved a probe approximately 20 cm from the seabed at a constant speed (1 m s^−1^) directly towards the burrow (figure 1*b*). The probe was designed as a novel threat and was made from a black plastic funnel with fluorescent flagging tape attached around the mouth of the funnel (figure 1*b*). This probe, in turn, was connected to a 2 m long PVC pole that was used by an experimenter to drive the probe forward during the simulated threat. The distance between the probe and burrow when the focal animals retreated (i.e. the FID) was marked with a small piece of coral by another observer (figure 1*b*). We then recorded, up to 20 min, the time taken for the goby and shrimp to re-emerge after the threat (i.e. the RET; figure 1*c*). Once the pair re-emerged from their burrow, we waited 2 min before repeating the entire process a further two times, resulting in three separate trials per each goby–shrimp pair. Water depth and time of the day were recorded at the start of each trial, while the FID was obtained after the completion of each trial by measuring, with a measuring tape, the distance between the deployed coral marker and the burrow. At the completion of all three trials, we estimated the size of the focal fish and shrimp (to the nearest 5 mm) using a ruler that was placed close to the burrow entrance for scale (figure 1*d*).

### (c) Statistical analysis

Data analysis was performed in *R* [27], version 3.5.3, using the packages *lmerTest* [28], *emmeans* [29], and *interactions* [30]. The significance level was set at *α <* 0.05.

We tested whether gobies differed in their willingness to re-emerge from their burrow (RET) after the simulated predator threat, and whether this variation was explained by variation in body size, FID, environmental conditions, and re-emergence behaviour of their mutualistic shrimp. Accordingly, we fitted a linear mixed-effects model with RET (seconds) as the dependent variable. Individual identities (random intercepts) were included in the random structure of the model to account for repeated measures, while the fixed effects were goby body size (length in cm), FID (cm), their interaction (size × FID), experimental day (1–4; consecutive days), time of the day (hours; continuous variable), trial (three repeated measures per pair; continuous variable), water depth (metres) and shrimp re-emergence during the trial (binary variable). The latter accounted for trials in which the goby emerged within the 20 min allocated for re-emergence, but the shrimp did not. All continuous variables (RET, body size, FID, time of the day, and water depth) were mean centred and scaled prior to the analyses (mean = 0; s.d. = 1) to aid in model fitting. Trial was also coded as numeric to test whether overall changes in the behaviour of animals happened over time.

We reduced the model complexity by removing covariates that were not essential for testing our hypothesis and that did not explain a significant portion of the behavioural variance observed. To do so, we used both likelihood ratio tests and Akaike information criteria to compare the full model, in which all fixed effects were present, with a null model, in which a fixed effect was excluded. The final model did not include day (ΔAIC = 3.350; *p* = 0.448) and water depth (ΔAIC = 1.810; *p* = 0.657). We verified the normality and homogeneity of the weighted residuals and ran pairwise comparisons with the *R* package *emmeans* [29], adjusted with the conservative Bonferroni method for significant categorical predictors (i.e. shrimp re-emergence during the trial; corrected *p* = 0.045), while accounting for the variation explained by other predictors.

Given the potential importance of body size in mediating antipredator responses of both species, we used a Pearson's correlation to test whether gobies and shrimp sharing a burrow were matched in body size.

We assayed 27 goby–shrimp pairs, which corresponded to as many pairs as we could logistically sample during the course of the study. Of these, 17 pairs contributed data for the analysis of antipredator responses (43 datapoints across trials) and 17 pairs for the Pearson's correlation test (see raw data and *R* code available at https://figshare.com/s/8a943f32f41c4d96adab). In the remaining replicates, either the goby or the shrimp did not re-emerge after the predator threat within the maximum 20 min allocated to RET or the assay had to be interrupted for logistical reasons (e.g. adverse weather). In addition, three pairs were excluded from the linear mixed-effects model entirely (CF5, CF7, and CF11), prior to analysis, because the accuracy of the behavioural data was compromised by adverse environmental conditions.

## 3. Results

**Figure 2.**
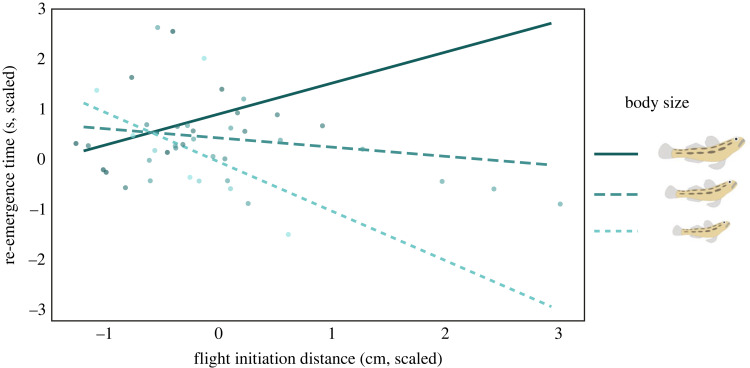
Interaction between the continuous predictors body size and flight initiation distance (FID, scaled) in the linear mixed-effects model with re-emergence time (RET, scaled) as the dependent variable. Intervals are calculated using false discovery rate adjusted (*t* = 2.36). FID slopes are represented separately for +1 SD (larger goby), mean (mid-sized goby), and –1 SD (smaller goby) body size. When body size is outside the interval [–0.15, 2.08], the FID slope is less than 0.05—the range of the observed values for body size is [–1.85, 2.45].

RET depended on goby body size. Specifically, after exposure to the simulated threat, larger gobies re-emerged later than smaller gobies (table 1). A significant portion of the variance in goby behaviour was explained by the interaction between body size and FID (table 1). In particular, the correlation between FID and RET was strongly negative in smaller (slope: −0.910; estimate ± s.e.: –0.980 ± 0.300; *p* < 0.001), absent in average-sized (slope: 0.131; estimate ± s.e.: –0.180 ± 0.160; *p* = 0.260), and had a marginally non-significant positive trend in larger gobies (slope: 1.170; estimate ± s.e.: 0.620 ± 0.340; *p* = 0.070; figure 2).

**Table 1.** Results from the model. Time of the day, trial (1–3), shrimp re-emergence (shrimp; binary variable), body size, flight initiation distance (FID), and their interaction (size × FID) are included as fixed effects in the model. Random intercepts are also included for each goby–shrimp pair, which allowed accounting for repeated measures. Analysis of variance was performed with Satterthwaite's method. Significance was *α* < 0.05 and significant results are in bold.

model					
RET					
fixed effects	estimate ± s.e.	mean sq.	d.f.	*F*	*p*
time of day	–468 ± 0.161	4.930	1,36	8.500	**0**.**006**
trial	–0.143 ± 0.152	0.517	1,36	0.892	0.351
shrimp	–0.649 ± 0.302	2.686	1,36	4.631	**0**.**0382**
body size	0.460 ± 0.120	3.073	1,36	5.298	**0**.**027**
FID	–0.284 ± 0.161	1.804	1,36	3.111	0.086
size × FID	0.770 ± 0.263	4.960	1,36	8.553	**0**.**006**
random effects	estimate ± s.e.				
among IDs	0.001 ± < 0.001				
within IDs	0.580 ± 0.185				

Gobies were consistent in their response to the perceived predatory threat across the trials: trial did not explain a significant portion of the variance (table 1). However, RET decreased during the day from morning to afternoon (table 1). The behaviour of the goby was also influenced by the behaviour of the shrimp (table 1), and *post-hoc* comparisons confirmed that gobies re-emerged sooner from the burrow when the shrimp also re-emerged during the trial (estimate ± s.e.: 0.649 ± 0.311; *d.f.* = 30.93; *p* = 0.045). There was a strong positive correlation between the body sizes of the gobies and shrimp (*r* = 0.422; *d.f.* = 49; *p* = 0.002), suggesting that individuals preferred to share a burrow with a partner that closely matched their body size.

## 4. Discussion

We investigated risk-sensitive behavioural responses in a goby–shrimp mutualism and found that body size influenced sandy-prawn gobies' antipredator behaviour. Specifically, after a simulated threat, re-emergence from the burrow was quicker in small than large fish. We also found that the relationship between FID and RET depended on goby body size: a faster re-emergence by small—but not medium- or large-sized gobies—was associated with an earlier flight from the approaching threat (i.e. when the threat was still further away). Fish were consistent in their behaviours across repeated trials but re-emerged sooner if tested later in the day. Moreover, we found that there was a close match in the size of gobies and shrimp sharing the same burrow, and that the re-emergence times of the two partners in this mutualism were highly dependent on one another.

Why was there a difference in the antipredator response of large and small gobies? Our finding that larger gobies were slower to re-emerge aligns with previous work in other taxa (e.g. [31]), including fishes. In three-spined sticklebacks, for example, refuge use presumably represents a balance between size-dependent metabolic demands and predation risk, with time spent hiding coming at a direct cost of lost feeding opportunities [14]. As a result, due to the metabolic costs associated with hiding, smaller fish may need to take more risks and this, in turn, could explain why they emerge sooner after a threat than their larger counterparts.

Based on the current literature, we expected a positive relationship between FID and latency to re-emerge: animals that flee sooner (i.e. when the predator is further away) should also hide longer [32,33], but see [31]. Here, too, body size seems to play a mediating role. Specifically, we found a positive—albeit marginally non-significant—relationship between these two antipredator responses only in larger-sized gobies. By contrast, we found a strong negative relationship between RET and FID in smaller gobies, in line with evidence on male fiddler crabs (*Uca lactea perplexa*) [31]. Here, the typically allometric relationship between body size and metabolic rate may, in turn, explain the negative correlation observed between FID and RET in smaller animals [34]. Specifically, given that smaller fish are more affected by lost feeding opportunities than larger fish [14], if fleeing sooner (i.e. having a longer FID) lowers the chance of detection, small individuals can then re-emerge more quickly to continue their activities. Thus, whether our results represent size-mediated differences in optimal antipredator behaviours is a question that warrants further investigation [35].

Intriguingly, we also found that gobies re-emerged sooner during the course of the day. It is not clear why this might be the case. One possibility is that this may be related to temporal shifts in the relative costs and benefits of remaining inside the burrow versus an earlier re-emergence. Such shifts could occur, for example, as a result of changes in hunger levels, energy reserves, mate availability, visibility or predation risk over the course of the day. We suggest that future studies may wish to explore these possibilities in more detail.

Our results, consistent with earlier natural history observations, suggest that gobies and shrimp are closely matched for size and that the antipredator behaviours of the two partners are closely coupled. The size-match between gobies and shrimp has been suggested to be driven by competition among fish for access to burrows, with smaller fish relegated to burrows built by smaller shrimp [36]. The fact that the antipredator behaviours are also closely correlated is consistent with the functionality of this partnership being crucial to survival [37]. The shrimp, which has poor eyesight, relies on the goby to act as a sentinel, warning of potential danger, with survival of the shrimp being greatly diminished in the absence of a goby partner. Two distinct, but non-mutually exclusive, processes might explain the coordinated antipredator behaviour observed within pairs: gobies share their burrow with shrimp that closely match their willingness to take risks (heterospecific assortment), and one partner in the relationship adjusts its response to conform to the other's antipredator behaviour (heterospecific conformity; [38]).

Taken together, our findings demonstrate how an individual's antipredator behaviour in the wild relates to its phenotype and other factors that affect the vulnerability it perceives, with mutualistic partners having similar risk sensitivities.

## Data Availability

All data, data description, and *R* code are provided in [39].
